# RETRACTION: Effect of Endoscopic Mucosal Resection and Endoscopic Submucosal Dissection on Postoperative Wound Complications in Patients With Gastric Cancer: A Meta‐Analysis

**DOI:** 10.1111/iwj.70356

**Published:** 2025-03-18

## Abstract

**RETRACTION**: LiuX. , WuX. , and FanW. , “Effect of Endoscopic Mucosal Resection and Endoscopic Submucosal Dissection on Postoperative Wound Complications in Patients with Gastric Cancer: A Meta‐Analysis,” International Wound Journal 21, no. 4 (2024): e14564, 10.1111/iwj.14564.38093697 PMC10961863

The above article, published online on 14 December 2023, in Wiley Online Library (http://onlinelibrary.wiley.com/), has been retracted by agreement between the journal Editor in Chief, Professor Keith Harding; and John Wiley & Sons Ltd. Following an investigation by the publisher, all parties have concluded that this article was accepted solely on the basis of a compromised peer review process. The editors have therefore decided to retract the article. The authors did not respond to our notice regarding the retraction.

**RETRACTION**: X. Liu , X. Wu , and W. Fan , “Effect of Endoscopic Mucosal Resection and Endoscopic Submucosal Dissection on Postoperative Wound Complications in Patients with Gastric Cancer: A Meta‐Analysis,” International Wound Journal 21, no. 4 (2024): e14564, 10.1111/iwj.14564.38093697 PMC10961863

The above article, published online on 14 December 2023, in Wiley Online Library (http://onlinelibrary.wiley.com/), has been retracted by agreement between the journal Editor in Chief, Professor Keith Harding; and John Wiley & Sons Ltd. Following an investigation by the publisher, all parties have concluded that this article was accepted solely on the basis of a compromised peer review process. The editors have therefore decided to retract the article. The authors did not respond to our notice regarding the retraction.

